# High Sensitivity SERS Substrate of a Few Nanometers Single-Layer Silver Thickness Fabricated by DC Magnetron Sputtering Technology

**DOI:** 10.3390/nano12162742

**Published:** 2022-08-10

**Authors:** Hsing-Yu Wu, Hung-Chun Lin, Guan-Yi Hung, Chi-Shun Tu, Ting-Yu Liu, Chung-Hung Hong, Guoyu Yu, Jin-Cherng Hsu

**Affiliations:** 1 System Manufacturing Center, National Chung-Shan Institute of Science and Technology, New Taipei City 237209, Taiwan; 2 Center for Astronomical Physics and Engineering, Department of Optics and Photonics, National Central University, Taoyuan City 320317, Taiwan; 3 Department of Physics, Fu Jen Catholic University, New Taipei City 242062, Taiwan; 4 Department of International Ph.D. Program in Innovative Technology of Biomedical Engineering and Medical Devices, Ming Chi University of Technology, New Taipei City 243303, Taiwan; 5 Department of Materials Engineering, Ming Chi University of Technology, New Taipei City 243303, Taiwan; 6 Kidney Research Center, Department of Nephrology, Chang Gung Memorial Hospital, College of Medicine, Chang Gung University, 5 Fu-Shing St., Taoyuan 33333, Taiwan; 7 Department of Engineering and Technology, School of Computing and Engineering, University of Huddersfield, Queensgate, Huddersfield HD1 3DH, UK; 8 Graduate Institute of Applied Science and Engineering, Fu Jen Catholic University, New Taipei City 242062, Taiwan

**Keywords:** surface-enhanced Raman scattering (SERS), silver nanoparticle (AgNP), rhodamine 6G (R6G), DC magnetron sputtering, SERS substrate, hotspot, analytical enhancement factor (AEF), limit of detection (LOD), relative standard deviation (RSD)

## Abstract

Surface-enhanced Raman spectroscopy (SERS) is commonly used for super-selective analysis through nanostructured silver layers in the environment, food quality, biomedicine, and materials science. To fabricate a high-sensitivity but a more accessible device of SERS, DC magnetron sputtering technology was used to realize high sensitivity, low cost, a stable deposition rate, and rapid mass production. This study investigated various thicknesses of a silver film ranging from 3.0 to 12.1 nm by field emission scanning electron microscope, X-ray diffraction, and X-ray photoelectron spectroscopy. In the rhodamine 6G (R6G) testing irradiated by a He-Ne laser beam, the analytical enhancement factor (AEF) of 9.35 × 10^8^, the limit of detection (LOD) of 10^−8^ M, and the relative standard deviation (RSD) of 1.61% were better than the other SERS substrates fabricated by the same DC sputtering process because the results showed that the 6 nm thickness silver layer had the highest sensitivity, stability, and lifetime. The paraquat and acetylcholine analytes were further investigated and high sensitivity was also achievable. The proposed SERS samples were evaluated and stored in a low humidity environment for up to forty weeks, and no spectrum attenuation could be detected. Soon, the proposed technology to fabricate high sensitivity, repeatability, and robust SERS substrate will be an optimized process technology in multiple applications.

## 1. Introduction

Since the development of Raman spectroscopy in 1928, its measurement spectrum has been applied to many research areas such as molecular vibrations [1], crystal structures [2,3], and its fingerprint-like specificity [4] to identify the specific chemical structure [5,6]. In 1974, British scientist Fleischmann discovered the surface-enhanced Raman spectroscopy (SERS) effect to improve the limitation of detecting the dilute molecule concentration Raman-active vibration signal amplified on a rough silver electrode bottom plate by a factor of 10^2^ to 10^4^. The most widely accepted SERS mechanism is mainly through electromagnetic enhancement (EM) and chemical enhancement (CE) cascade processes. The local EM is radiated from the incident light scattered from the nanostructured metallic substrate. The EM effect is in the proximity of the nanostructured surfaces, where coherent electrons oscillate around the surfaces or nanoscale crevices to excite the molecule’s localized surface plasmon resonance (SPR). The EM coupling of the oscillating dipoles from the adsorbed analytes can mainly contribute to Raman enhancement on the metal surfaces [7]. Therefore, the SERS enhancement factor (EF), defined as the product of EM and CE, is described as EF = EM × CE. The EM effect can generally reach 10^2^–10^3^ times and is more robust than CE [8]. In most cases, the weaker CE spectral signals are amplified through the EM effect. Therefore, the EM effect is a significant factor in influencing SERS sensitivity.

About ten years after the discovery of SERS, the discovered SERS hotspots with silver particle spacing play a dominant role in enhancing the intrinsic Raman signal of the target molecule [9]. The inter-particle distance in silver nano-island arrays of less than 10 nm is crucial for the enhancement [10,11]. Moskovits et al., concluded that the light scattered from the molecules was amplified further if struck on nanostructured gold or silver surfaces appropriately. The nanoscale of clefts, gaps, and fissures in nanostructured metals forming the “hotspot” can accumulate incoming light and concentrate the electromagnetic energy to function as an antenna to collect radio waves [12].

Since then, SERS has been a powerful method for analyzing chemical and biological species down to single molecules on surfaces or in solutions [1,2,3,4,5]. The presence of individual analyte molecules can be detected because the relevant characteristic signals are enhanced. SERS is an effective fingerprint analysis technique for the real-time field detection of biological and chemical sensing due to its favorable non-destructive and non-invasive properties [13]. Nowadays, SERS has the characteristics of selectivity, rapidity, simplicity, and specificity, and has been widely used in the fields of molecular identification [1], drug monitoring, medical diagnosis, biological detection [5,14], water contaminants [15,16], health care [17,18], food safety [19,20], biochemical and medical analysis [21], and bacteria [22].

Another challenging issue of SERS substrates is that the substrates quickly lose their sensitivity and stability when the device is stored in the atmosphere [23]. A wet-chemical synthesis of nanostructures is a technical method to improve atmospheric stability [24,25,26]. However, the synthesis method is time-consuming and not reproducible for the optimal surface morphology to yield maximum SERS enhancement. To solve this problem, Sha et al., deposited a silver nano-island array layer by electron beam evaporation and a 20 nm thick Au shell by magnetron sputtering on an Ag@Au core-shell SERS substrate. This method can have good activity in storing a vacuum package [27,28] since the weaker scattering enhancement Au layer on its top acts as a protective layer to anti-oxidation and attenuates the optical background noise. Moreover, the characteristics of the plasmonic fields realize the deposition process based on the nanoparticle shape, and therefore multiple designs of nanostructured platforms have been innovatively developed to amplify the Raman response. For example, Minopoli et al., found that nano-spheres and nano-bars exhibited better SERS efficiencies at a surface coverage broader than 50% due to the higher number of plasmonic hotspots [29].

This study reported a reliable technology to fabricate the SERS substrate of a single silver layer using a DC magnetron sputtering method. It is relatively difficult to fabricate a silver layer using thermal evaporation due to controlling the nanoscale thickness and RF sputtering due to the low deposition rate in a low DC bias voltage during deposition. Furthermore, compared to the Raman enhancement affected by different layer thicknesses and hotspot regions, the proposed technique deposited nano-grain layers, forming a more uncomplicated structure to manufacture a SERS substrate. This illustrated the hotspot volume of the SERS substrate by depositing different nanoscale silver thicknesses because of the SERS EM effect produced in the proximity of the silver surfaces or nanoscale crevices. The acknowledgment of optimum thickness will benefit the academic development of the SERS enhancement.

The results show that dilute concentrations of the R6G analyte in 10^−4^–10^−7^ M detected with varying silver monolayer thicknesses of SERS ranging from 3.0 to 12.1 nm. Further analysis showed that the produced SERS substrates had high sensitivity and low bias when the devices were evaluated by scanning electron microscopy morphology, X-ray diffraction, X-ray photoelectron spectroscopy (XPS), and Raman spectroscopy. The SERS substrate can greatly enhance the SERS signals of other molecules such as paraquat and acetylcholine, and the testing proved that this new SERS device had high sensitivity. In addition, long-lifetime SERS substrates have been investigated that can be stored in a vacuum desiccator or moisture-proof cabinet for up to 40 weeks without losing their sensitivity. All of the optimized analyzed data indicate that the SERS technique proposed in this paper can be a candidate stratagem to manufacture the SERS substrate for many applications in the near future. This proposed technology will be a candidate method to realize a robust, low-cost, stable deposition rate, and high mass production rate.

## 2. Materials and Methods

**Figure 1:**
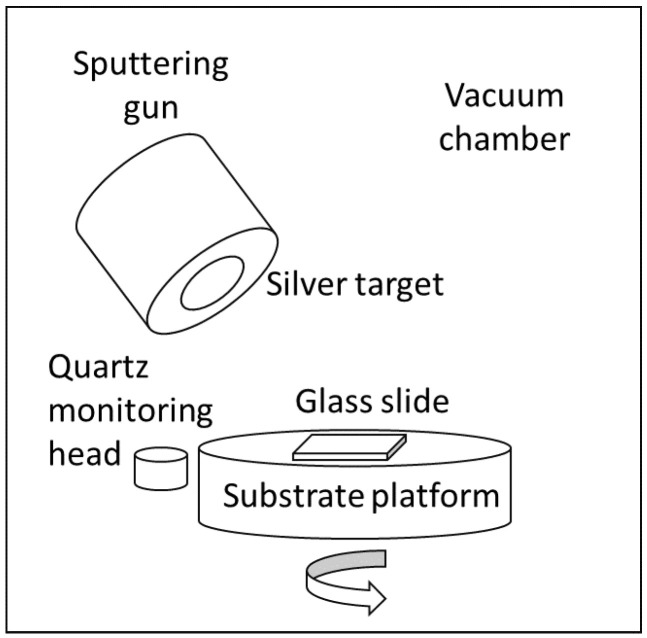
The schematic diagram of the sputtering system.

The glass slide dimensions of 76.2 × 25.4 × 1 mm were used as the SERS substrate. For the experimental preparation, the substrates were polished with wet cotton, moistened with CeO_2_ powder, and then ultrasonically cleaned for 20 min. After, the substrates were blown with clean nitrogen gas and then settled in a substrate platform of a vacuum chamber, as shown in Figure 1. The volume of the vacuum chamber was 45 cm in diameter and 50 cm in height. Then, a 5 cm diameter sputtered target loaded with 99.99% metallic silver was mounted approximately 150 mm above the substrate on the sputter gun. In addition, the quartz crystal monitoring head was located near the substrate platform to control a silver thin film.

When conducting the experiments, a diffusion pump drew the base pressure of the chamber to 10^−5^ torr. During the deposition process, the working pressure was kept at 3 × 10^−3^ torr when the working argon gas (99.995%) was fed into the chamber at about ten sccm. In this case, the parameters of the DC generator (PFG 1500 DC, made by Huttinger Electronic, Inc. Freiburg, Germany) were 316 V voltage and 0.01 A current. The substrate rotation speed was 30 rpm to maintain a uniform thickness of the silver layer. The significant tooling factor is the ratio of actual thickness at a substrate to thickness reading on display. The method to calibrate this factor was to deposit a silver layer of about 284 nm thick in about 7402 s. This process can achieve a relatively stable deposition rate of 2.30 ± 0.02 nm/min, monitored by a quartz crystal monitor, and the deposited time for each SERS substrate is discussed in Section 3.1.

To evaluate the sensitivity of the SERS device, six different silver thicknesses, labeled as Samples A to F, and their deposition times were prepared and are summarized in Table 1. Before conducting the following examinations, these samples were stored in a moisture-proof cabinet with 25% humidity, a vacuum desiccator, and an air environment. First, a field-emission scanning electron microscope (FESEM, JSM-7600F, Jeol, Tokyo, Japan) was used to analyze the morphologies and cross-sections of the SERS substrates. The as-deposited samples were then characterized by an X-ray diffractometer (Bruker D8 Advance) with Cu-Kalpha radiation (λ = 0.15406 nm). Finally, an XPS (ULVAC-PHI, Inc., PHI 5000 VersaProbe III, Kanagawa, Japan) was applied to analyze the chemical bonding state at the surface of the silver layer.

In the aqueous solution testing, the R6G, paraquat, and acetylcholine analytes were dropped onto the AgNP layer of the SERS substrates. The Raman spectroscopy (HR Evolution, Horiba, Kyoto, Japan), applied with a 0.1 mW He-Ne laser beam focused by a 100× objective lens (NA 0.9), measured the Raman spectrum ranging from 400 to 2000 cm^−1^.

## 3. Results

### 3.1. Effect of Sputtering Time on Surface Morphologies of the Silver Layers

**Figure 2:**
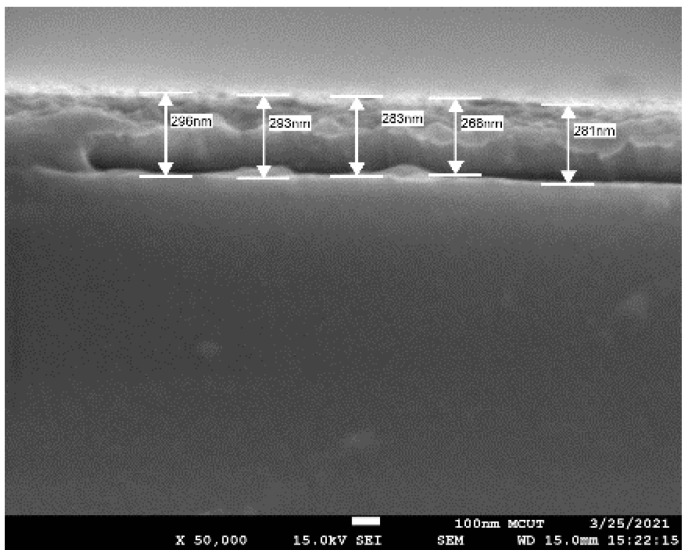
The FESEM cross-sections of the deposited silver layer to calculate the tooling factor.

The calibrating tooling factor is a fundamental process to produce the required thin film thickness for target sputtered materials. The process is to deposit through a 150 ± 10% tooling factor based on the quartz crystal monitor display. The silver layer with an average 284 ± 3 nm cross-section, as shown in Figure 2, was then evaluated by FESEM, and this value was used to adjust the crystal monitor display. Hence, the deposition rate of 2.30 ± 0.02 nm/min was derived from the FESEM thicknesses divided by the deposition time of 7402 s. The deposition times from Samples A to F are reported in Table 1.

**Figure 3:**
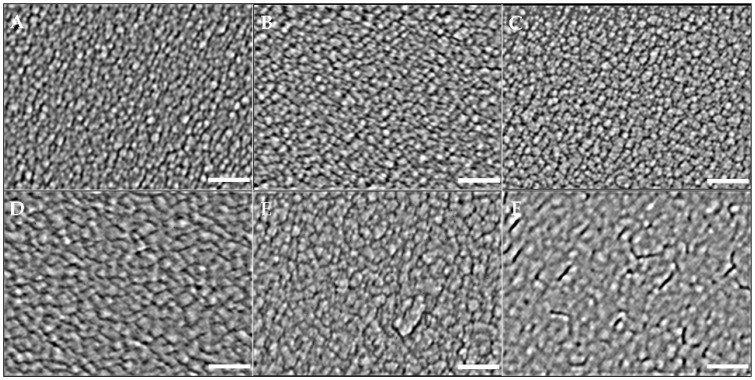
Images of the FESEM morphologies using the magnification of ×200,000 to analyze Samples (**A**–**E**). The size of the white bars in each sub-image is 100 nm. Samples (**A**–**E**) show the nanoparticle structures, and Sample (**F**) shows a liquid-like structure.

Figure 3 shows the FESEM morphologies of the silver films deposited at their relative times according to Table 1. The silver nanoparticles of Samples A and B are transparent. When increasing the deposition time, the nanoparticles grew such as Samples C, D, and E. The resulting thickness of Sample F formed a liquid-like structure.

**Figure 4:**
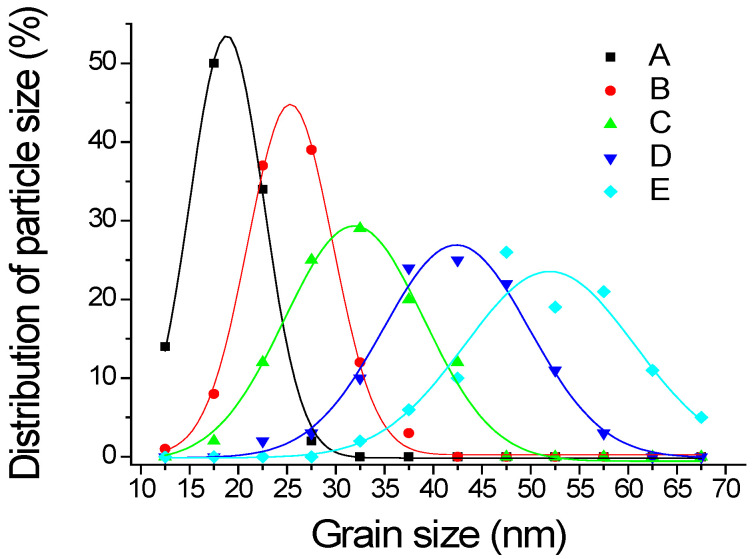
The grain sizes of Samples (**A**–**E**) are nearly Gaussian distributions.

To further analyze the sizes of the nanoparticles and hotspots, ImageJ software provided by the U.S. National Health Institute was applied to analyze the morphological features and quantify the complexity of the digital images [30]. The particle sizes of Samples A–E are shown in Figure 4 and are summarized in Table 2, where the maximum value ± FWHM/2 represents the sizes, and FWHM is the full width half maximum intensity of the Gaussian distribution. Sample A had the smallest grain of 18.8 ± 4.5 nm, and Sample *E* had the largest grain of 51.9 ± 10.2 nm. When evaluating the dimension of the hotspot within the five samples, 50 locations per sample were randomly selected and the resulting average size was approximately 5.3 nm. Sample F could not be evaluated due to the aggregation of the layer.

### 3.2. XRD of AgNP Structures

**Figure 5:**
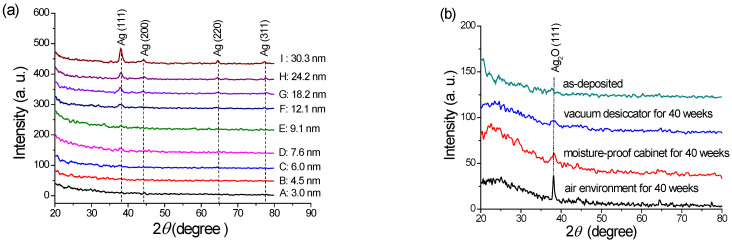
(**a**) The XRD patterns of the AgNP layers (the as-deposited Sample A to I) with various thicknesses. (**b**) The XRD measurements of Sample C from the as-deposited, stored in a moisture-proof cabinet, vacuum desiccator, and air environment for up to forty weeks.

Each sample was stored in a vacuum desiccator to prevent silver oxidation when the SERS substrates completed their deposition process. The XRD and XPS were then used to examine the degree of oxidation between Samples A–I with the relative silver thickness of 3.0, 4.5, 6.0, 7.6, 9.1, 12.1, 18.2, 24.2, and 30.3 nm, as shown in Figure 5a. The measured XRD peaks at the values of 2*θ* were 38.0, 44.2, 64.5, and 78.3°, corresponding to the crystal face structures of Ag(111), Ag(200), Ag(220), and Ag(311), respectively. For Sample I, the apparent polycrystallinity of the Ag(111) phase and Ag(200) was caused by the thicker silver layer. The face-centered cubic (FCC) crystalline phase measurements were identified as Ag ICDD (International Center for Diffraction) card number 00-004-0783. Figure 5b shows the characteristic diffraction peak of Ag_2_O(111) at 2*θ* = 38.0°, which agrees with the Ag(I) oxide FCC crystalline phase (JCPDS 041-1104) studied by Rajabi et al. [31], associated with the different storage of Sample C for up to forty weeks. The sample storage in the air environment displayed a clear peak because the humidity affected the structure.

Each crystalline size of the nanoparticles was determined from the FWHM of the X-ray diffraction pattern according to the dominated direction (111) based on the Scherrer equation [32], described as:

*D* = 0.09λ/*B* cosθ, (1)

where *D* is the crystallite size; λ is the wavelength of 0.15406 nm using Cu-kα radiation; *B* is FWHM; and θ is the Bragg angle. However, measuring the crystallite-scale dimensions of Samples A–E was difficult because the samples were too thin to measure. To evaluate the nanoscale structures of Samples A–E, an indirect method of the thicker the layer, the larger the crystallite scale sizes, was adopted to realize their structures, as shown in Samples F–I in Table 3.

### 3.3. X-ray Photoelectron Spectroscopy of AgNP Structures

**Figure 6:**
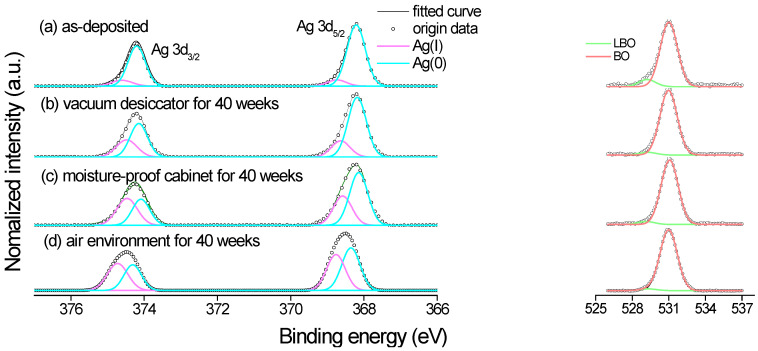
The high-resolution X-ray photoemission spectroscopy scanning from the Ag 3d and O 1s regions.

According to Sample C of the optimal SERS substrate discussed in Section 3.4, Sample C was selected to be examined by X-ray photoelectron spectroscopy (XPS), in which the binding energy was calibrated by C 1s (284.6 eV). The measurement of the Ag 3d spectrum of AgNPs from high-resolution XPS to evaluate the environmental test is shown in Figure 6. Two peaks centered at ~368.1 and ~374.1 eV can be attributed to the binding energies of Ag 3d_5/2_ and Ag 3d_3/2_, with a splitting energy of 6.0 eV. The FWHMs of the 3d_5/2_ and 3d_3/2_ peaks of the as-deposited sample were 0.628 and 0.613 eV, respectively. The 3d_5/2_ peak was further analyzed to show a significant peak of Ag(0) located at 368.2 eV and a fainter peak of Ag(I), which was Ag-O-, located at 368.7 eV [33]. Table 4 shows the surface relative atomic concentrations of Ag(0) and Ag(I) elements in Sample C stored in the different environments. The O 1s XPS spectra also contained two components, the peak at 531.0 eV with the most significant border area for bridging oxygen (BO) and fainter peaks at 529.2 eV for low-binding-energy oxygen (LBO) [34,35].

### 3.4. Using the SERS Samples to Detect R6G

**Figure 7:**
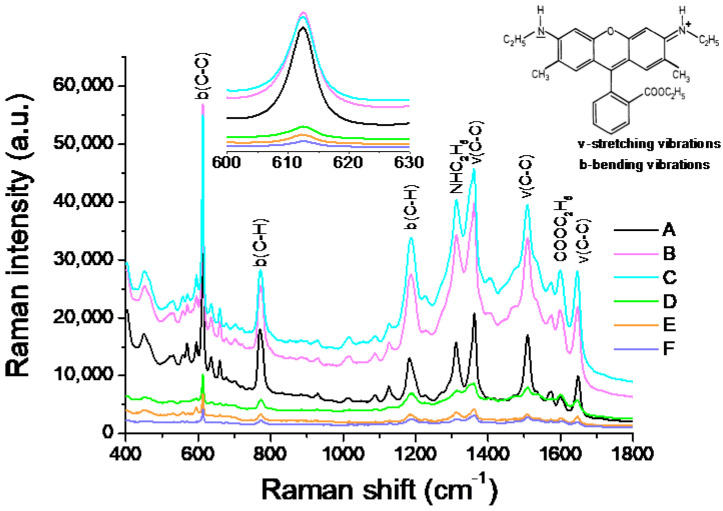
The SERS spectra resulting from the R6G analyte (10^−6^ M) by the silver layers of the various relative thicknesses.

Typical detectable Raman-active modes of R6G probes are summarized in Table 5. Samples A–F deposited with their relative silver thicknesses were used to detect the R6G of 10^−6^ M, and the Raman spectra are summarized and illustrated in Figure 7. Many apparent peaks illustrated the signals of the bindings around the R6G molecular.

**Figure 8:**
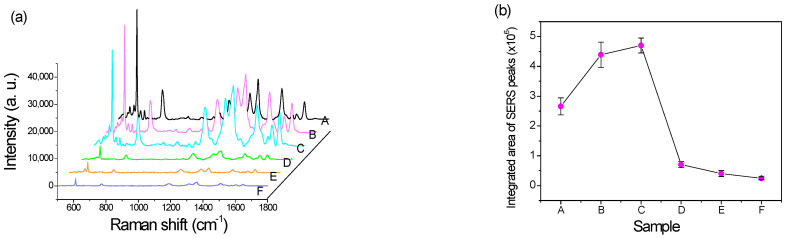
(**a**) The background-subtracted SERS spectra and (**b**) their integrated intensities of the 10^−6^ M R6G analyte on different thicknesses of the Ag-deposited substrate.

As shown in the sub-picture in Figure 7, ranging from 600 to 630 cm^−1^, the Raman signals and background noises of Samples B and C were relatively higher than those of Sample A. That is, their Raman enhancement increased with the silver layer thickness. As the spectra of Figure 7 enveloped the enhancement signal and successive background noise, the signal enhancement could be evaluated by the signal-to-background noise ratio (S/B ratio), as shown in Table 6. Sample A seemed to receive the largest signal. It is essential to further analyze the sensitivity among Samples A–C. Figure 8a shows the background-subtracted SERS spectra of the 10^−6^ M R6G analyte on the as-deposited silver substrate with different thicknesses. Sample C illustrates the optimal SERS enhancement because all of its SERS peaks, except at 612 cm^−1^, were higher than those of the other samples.

To this end, the integrated area of the background-subtracted SERS spectra was conducted by referring to the layer thickness as summarized in Figure 8b. The integrated intensity increased with the layer’s thickness until 6 nm (Sample C), then profoundly decreased at 7.6 nm (Sample D), and fell to the lowest intensity of 12.1 nm (Sample F). Hence, even though the S/B ratio from Sample A was larger than Sample C, as listed in Table 6, Sample C presented the optimal SERS enhancement due to the highest integrated intensity of the SERS spectrum.

**Figure 9:**
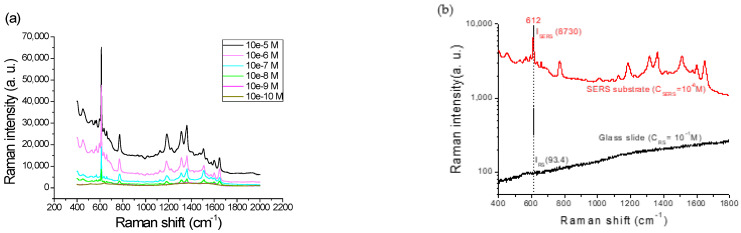
(**a**) The SERS intensities with R6G concentration between 10^−5^ to10^−10^ M in Sample C. The limit of detection (LOD) observed was lower at 10^−8^ M. (**b**) A comparison of the Raman spectra between Sample C for measuring the 10^−6^ M R6G analyte and a pure glass slide for 10^−1^ M.

In Figure 9a, the various R6G of concentrations from 10^−5^ to 10^−10^ M were used to determine the sensitivity of Sample C, in which the limit of detection (LOD) was 10^−8^ M and the analytical enhancement factor (AEF) was 9.35 × 10^8^, as evaluated at 612 cm^−1^ by the following formula:

AEF = (I_SERS_/C_SERS_)/(I_RS_/C_RS_), (2)

where I_SERS_ (8730) is the measured intensity of the characteristic molecular signal by the SERS substrate in concentration C_SERS_ (10^−8^ M); I_RS_ (93.4) is the intensity by a glass slide in concentration C_RS_ (10^−1^ M), as shown in Figure 9b.

### 3.5. Reproducibility and Storage of SERS Substrates

**Figure 10:**
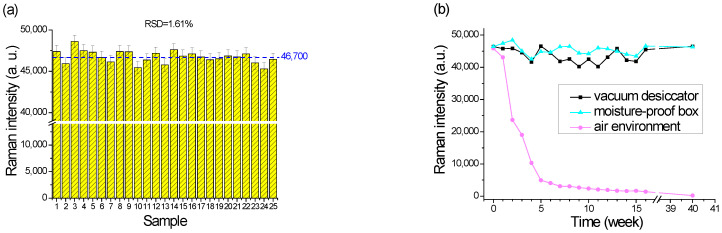
(**a**) The reproducibility of the SERS substrate evaluated by a 10^−6^ M concentration of R6G. (**b**) Lifetime comparisons of the SERS Sample C storage up to forty weeks in a vacuum desiccator, a moisture-proof cabinet, and an air environment.

Reproducible, reliable efficacy, and performance signals are other essential factors to evaluate the SERS substrates. Hence, five random samples of Sample C were dropped in a 10^−6^ M aqueous R6G solution on five random positions of each substrate to check the repeatability and sensitivity. Figure 10a shows all of the 25 SERS intensities from 610 to 615 cm^−1^. The average intensity and the RSD value for Sample C were 46,700 and 1.61%, respectively.

Another important factor in determining a robust SERS substrate is to evaluate its lifetime. Figure 10b shows the Raman intensities of another 54 prepared SERS substrates for Sample C, which were separated into three groups to be stored in a vacuum desiccator, a moisture-proof cabinet, and an air environment for up to forty weeks. The samples stored in the vacuum desiccator or moisture-proof cabinet achieved excellent quality. The average SERS intensities of the samples separately stored in the vacuum desiccator and the moisture-proof cabinet were 43,600 ± 5% and 45,400 ± 3.3% for forty weeks. Only the samples stored in the air environment reduced the intensity to 5000 strength after five weeks.

### 3.6. AEF Evaluated by Other Analytes

**Figure 11:**
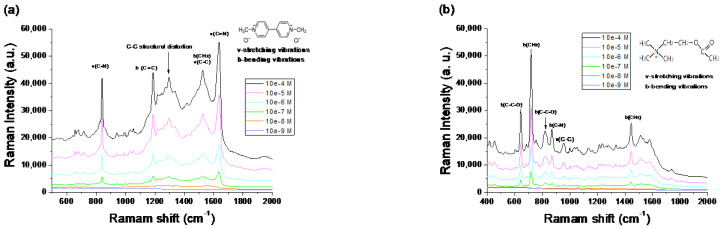
The Raman spectra from the SERS Sample C with various (**a**) paraquat and (**b**) acetylcholine analyte concentrations.

Sample C was further used to evaluate the sensitivity in other analytes. Figure 11a,b shows the excellent performance of the testing in paraquat (e1,1′-dimethyl-4,4′-bipyridinium dichloride, (C_6_H_7_N)_2_Cl_2_)) and acetylcholine (CH_3_OCOCH_2_CH_2_N^+^-(CH_3_)_3_), respectively. Paraquat is a highly toxic herbicide that can easily cause poisoning and death to humans and animals [38]. The chemical transmitter acetylcholine is a specific substance that induces nerve stimulation diseases [39]. From the experiments, the proposed SERS substrates can detect paraquat and acetylcholine with the 10^−7^ M LOD values of 3.19 × 10^7^ and 7.5 × 10^7^ AEF values, respectively.

## 4. Discussion

### 4.1. Effect of Sputtering Time on Surface Morphologies of Thin Silver Layers

As discussed in Table 2, the AgNP sizes of the sputtered layers from 18.8 nm to 51.9 nm were related to the processed deposition time. From Samples A to E, the longer the deposition time, the larger the grain size. By further increasing the thickness, as Sample F shows in Figure 3, a liquid-like coalescence began to form on the substrate, since the injected silver particles changed the morphology of the layer, resulting from the lateral growth of the nanoparticles in the longer sputtering time [40]. The processes included the migration of single adatoms on the substrate surface, the aggregation of the adatoms, nucleation to form nanoparticles, the growth and coalescence of nanoparticles to form a liquid-like structure, and continuous layer formation are associated with the Volmer–Weber growth of high-mobility metal films [41,42].

Table 2 shows the average sizes of the hotspots maintained at about 5.3 nm because of the metallic self-assembly during deposition. As Rastogi et al., reported, the gold nanoparticle cluster arrays exhibited less than 10 nm wide inter-cluster hotspots produced on glass [43]. During the deposition, the hotspot physically adsorbed silver vapor atoms, which might move across the surface by hopping from one potential well to another silver adatom, assisted by the thermal activation from the surface energy and kinetic energy parallel to the surface. Within the limited residence time at the position of an atom, the atom interacted with the other adatoms and incorporated the surface to form the stable nano-grains by releasing condensation heat to less surface energy [44].

Until the thickness of the AgNP layer increased to 12.1 nm, the nano-grains agglomerated to the liquid-like structure, as shown in Sample F in Figure 3, and the number of hotspots further decreased. The continuously deposited silver atoms diffused toward the existing grains on the substrate until the spaces of the hotspots were filled and formed a continuous thin film. The SERS intensity of each sample shown in Figure 7 and Figure 8a was related to the AgNP thickness. Hence, the deposited silver layer that was 6 nm thick was evaluated as the largest number of hotspots due to the highest SERS sensitivity. This assumption is discussed in Section 4.4.

### 4.2. XRD of AgNP Structures

X-ray diffraction (XRD) was used to discover the formation and crystal structure of the silver oxide phases that reacted with oxygen during the deposition process or in the storage environment. Al-Sarraj et al., indicated the X-ray diffraction peaks of a silver-oxide film at 2θ = 32.3° and 37.3°, corresponding to Ag_2_O (111) and Ag_2_O (200). The AgO was at 2θ = 34.1° and the mixed-phase Ag/Ag_2_O formation was 2θ = 39.6° [45]. In our study, no significant intensities in the AgO and Ag_2_O peaks, as shown in Figure 5a, might indicate the high purity silver structure of the stored samples, as studied by Waterhouse et al. [46]. The small peaks of Ag(220) and Ag(311) also appear, as Shen et al., studied using the XPS pattern and Asanithi et al., studied with selected-area electron diffraction (SAED) [41,47]. The XPS measurements that demonstrated the silver oxidation are discussed in the following section.

However, Samples A–E expressed unapparent XRD diffraction patterns in Figure 5a. Sagara et al., also claimed that the silver crystallite size of 30 to 40 nm could be realized at a 150 nm thickness of the silver thin film deposited by applying the sputtering voltage of 500–600 V at 2 and 4 mtorr [48]. In this study, small peaks of Ag(111) and the thicknesses of the layers less than 30.3 nm (Table 3) were measured at 2*θ* = 38°, and the crystalline sizes were less than 10.78 nm. Moreover, the more significant the crystallite size and the peak, the thicker the deposited thickness. Furthermore, the diffraction peak of Sample C, shown in Figure 5b, clearly appeared when stored in an air surrounding environment for forty weeks. The structure could be Ag_2_O(111) due to the limitation of current technology in detecting the Ag(111) peak. However, the structure was only Ag_2_O(111) because the deposited sample had no obvious Ag(111) peak, and the thickness of the silver layer did not increase automatically. Simultaneously, the translucent light purple of the as-deposited sample changed to translucent light yellow due to an oxidation reaction from the humidity. Some Ag_2_O (111) peaks were found even in moisture-proof or vacuum samples because the humidity intrusion could not be wholly prevented by moisture-proof or vacuum storage. The following section discusses the oxidation reaction to form the Ag_2_O phase.

### 4.3. X-ray Photoelectron Spectroscopy of AgNP Structures

X-ray photoelectron spectroscopy (XPS) is a powerful technique to measure the surface of a thin film less than 10 nm in thickness. Its qualitative and quantitative results can be used to examine the oxidation state by the photoelectron analysis. It has been reported that only about a 1.2 eV interval exists in spectral features between the Ag 3d_5/2_ binding energies of metallic silver and its oxides. That is, Ag 3d_5/2_ is calculated from 367.9 to 368.4 eV for Ag(0) and from 367.6 to 368.5 eV for Ag_2_O [49]. The theoretical calculation shows that Ag(I) and Ag(III) are more stable than Ag(II) [50] because most silver oxides, AgO_x_, x > 1, are potent oxidants [51]. The binding-energy intervals of Ag(0), Ag(I), and Ag(III) overlap with each other.

In this study, however, the XPS spectroscopy presented the simple Ag 3d binding energies of Sample C stored in the four different environments. All of the spectra of the samples only consisted of Ag 3d_5/2_ and Ag 3d_3/2_, as shown in Figure 6. The FWHM for the Ag 3d_5/2_ peaked only in the region of 0.628 to 0.939 eV in Table 4. The percentage compositions of the Ag(0) and Ag(I) elements were approximately 90.7% and 9.3%, respectively, indicating that most of the pure silver element Ag(0) existed in the as-deposited Sample *C*. However, Ag(0) was somewhat decreased to 78.4%, 64.1%, and 53.9% when separately stored in the vacuum desiccator, the moisture-proof cabinet, and the air environment. The enhancement of the O 1s peak intensity indicated the oxidation increase and the oxygen found in AgNP was different from the oxygen in the silver oxides [52], as shown in Figure 6. The prominent BO peak at 531.0 eV represented the hydroxide of water molecules from the humidity adsorbed on silver particles [53]; the smaller LBO peak at 529.2 eV represented the Ag_2_O due to the absorbed oxygen by the silver [52]. This phenomenon illustrates the apparent humidity intrusion of the AgNP layer stored in an air environment.

### 4.4. SERS Detection of R6G

Figure 8b shows the integrated intensities of the background-subtracted SERS spectra. Sample C was determined to be the most sensitive among the tested samples, resulting from the optimal silver thickness having a greater quantity of hotspots. Sample C featured the largest contacted surface areas to react with the aqueous solution of the R6G analytes to induce the highest SERS signal enhancement. Compared to the AgNP structures shown in Figure 3, Sample F significantly reduced the SERS enhancement because the continuous thicker silver structure had fewer hotspots. It was concluded that the thickness and structure of the AgNP layer indeed affect the SERS signal.

**Figure 12:**
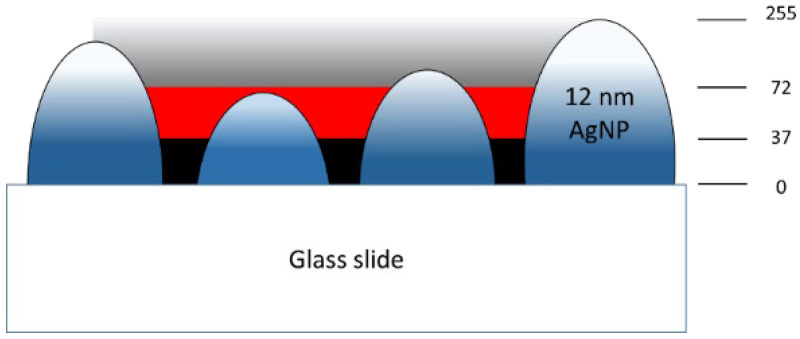
A schematic diagram of the hotspot region in the thickness direction on the Ag-deposited SERS substrate.

**Figure 13:**
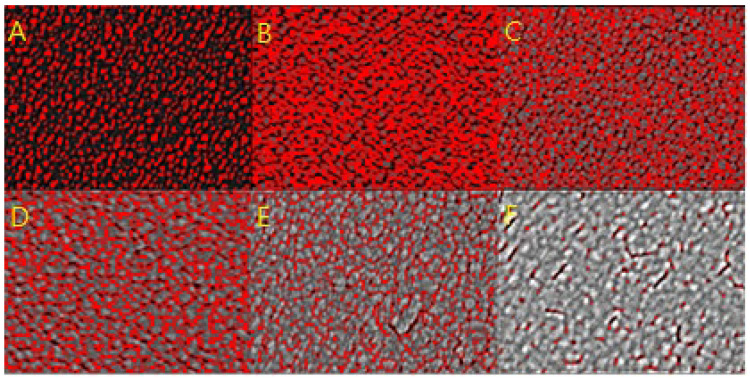
The different hotspot areas on Sample (**A**–**F**) with the different Ag-deposited thicknesses.

Accordingly, the cross-section of the AgNP layer and its relatively active hotspot region is sketched in Figure 12, where the relative greyscales are referenced to various Ag thicknesses from 3.0 nm for Sample A to 12.1 nm for Sample F. For example, the average brightness of Sample A was lower than that of Sample F as it was thinner. Then, the images of the FESEM morphologies in Figure 3 were converted to 0–255 greyscale to represent its three-dimensional structure. Hotspot regions were determined and marked in red between 37 and 72 grayscales, referring to the integrated SERS intensities from the maximum value of 4.7 × 10^6^ to the minimum value of 2.5 × 10^5^ in Figure 8b. The grayscale of the other thickness less than 37 was marked in black, and that larger than 72 in gradient-grey. This principle was used to transform the FESEM morphology images in Figure 13 to illustrate the hotspot regions according to the thickness of the layer.

In Figure 13, the red area is the existing hotspot within the thickness between 1.8 ± 0.9 nm and 3.4 ± 1.7 nm of the thickness of the silver layer. Within this, the thickness below 1.8 nm is illustrated as the black area, and above 3.4 nm as the gradient-grey area. Zhang et al., also support the inference that hotspots, with their density and along the *z*-axis in SERS substrates (i.e., increasing the Ag-deposited thickness), can affect the excitation hotspot volume directly [54]. Accordingly, the hotspot volumes for Samples A–C were the hotspot area estimated using the ImageJ software multiplied by the thickness of the silver. However, there can be two types of groove spacing to limit the enhancement of the SERS signal. One is the bottom of the groove spacing without the deposited silver. The bottom area marked in black had fewer hotspots to react with the R6G analyte in the aqueous solution such as Sample A, as shown in Figure 13. The other type is the top area of the groove spacings on the thicker silver layers. The spacings in the top area marked in gradient-gray, as shown in Sample E, also had fewer hotspots because their dimensions were larger than the hotspot spacing, where the EM between the spacings was too weak to produce the SERS enhancement. In the other case, the thickest, Sample F, only had a smaller effective thickness to become a small hotspot volume because the R6G analyte had difficulty entering the narrow and deep grooves of the coalesced silver layer due to the cohesion of the solution.

**Figure 14:**
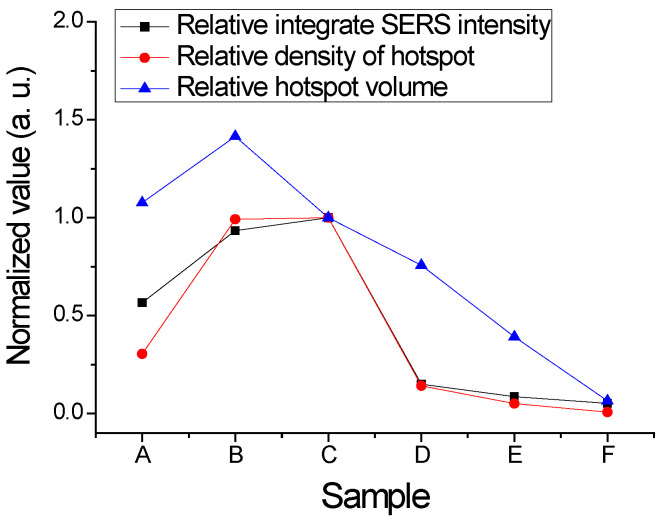
The relative densities of hotspots in the AgNP samples.

Therefore, the hotspot density varied with the thickness of the silver. Figure 14 shows the relative hotspot volume normalized for Sample C. The volume of Sample B was the largest among the samples. The relative densities of hotspots were the products of the volumes and the relative integrated SERS intensities normalized by the data in Figure 8b. Sample C still had the largest density of the hotspot. The standard deviation between the densities and SERS intensities was only 0.11. That is, the hotspot volumes were expected, as we predicted.

In addition to the surface enhancement that occurred in the hotspot volume, electromagnetic and chemical enhancements of the SERS are other important conditional factors [8]. The optimal Sample *C* detection had a LOD value of 10^−8^ M, quantified with the SERS intensity at the R6G concentrations between 10^−5^ and 10^−10^ M, as shown in Figure 9. The LOD value was compared to those of several R6G substrates deposited by the same sputtering technique in several studies over the past three years, as shown in Table 7. Among these published studies, the method of modifying AgNPs on a ferronickel (NiFe) alloy and the cicada wing had a small LODS of 10^−7^ M. In our new proposed strategy, sputtering the 6 nm silver layer on a cost-effective commercial glass slide could decrease the LODs to 10^−8^ M.

### 4.5. Reproducibility and Storage of SERS Substrate

Following the optimized SERS substrate of Sample C, an AEF of 9.35 × 10^8^ was achieved as assessed by dropping the R6G analyte in a concentration of 10^−8^ M, as shown in Figure 9b. Then, more SERS samples were prepared by dropping in 10^−6^ M to demonstrate their excellent reproducibility, with a high intensity of 46,700 and 1.61% RSD, as shown in Figure 10a. The average intensity of the substrates stored in a moisture-proof cabinet for up to forty weeks achieved 45,400 ± 3.3%, which was the same as the as-deposited sample, as illustrated in Figure 10b. That is, the service life of the substrate was more than forty weeks. Moreover, the SERS substrate remained more sensitive during storage because the moisture-proof cabinet with 25% humidity could dehumidify the substrate surface to prevent moisture intrusion.

### 4.6. AEF of Different Analytes: Paraquat and Acetylcholine

To evaluate the ability of the SERS substrates used to detect other analytes, the AEF values of the paraquat and acetylcholine analytes in a concentration of 10^−6^ M were 3.19 × 10^7^ and 7.5 × 10^7^, respectively, and their LOD values were in 10^−7^ M, as shown in Figure 11a,b. Although the AEF values were not outstanding in the SERS substrates fabricated by a chemical method, they have already performed well for physically manufactured substrates. Moreover, the SERS sample manufactured by the DC sputtered technology has promising analyte spectroscopy detection capability and reproducibility. In addition, the short time needed for the processing method can achieve better stable film thickness control and surface topography than the chemical fabricated SERS substrate. The DC sputtering method to manufacture SERS substrates can be used to realize low-cost and high-volume production. Using this technology to prepare a SERS substrate is expected to achieve high potential applications in real-life such as in agriculture, aquaculture, pastoralism, and mixed contaminants in drinking water and to help in the detection of drug hazards and abuse.

## 5. Conclusions

In this study, we successfully prepared silver nanoparticles (AgNPs) with different-sized particles on glass slides for SERS detection by controlling the deposition time of the DC sputtering. The SEM images show that the thickness and grain size of the films increased with the deposition time. We predict that hotspots can be generated by the DC sputtering between the silver layer thicknesses of 1.8 ± 0.9 nm and 3.4 ± 1.7 nm. Regardless of the deposited thickness of the layers, the hotspot spacing barely changed at about 5.3 nm. The optimal deposited silver thickness on a glass slide was about 6 nm, and the grain size of the AgNPs was about 31.9 nm. The as-deposited layer investigated by XRD and XPS was a pure silver material, which could enhance the SERS intensity of the dilute R6G analyte with the AEF of 9.35 × 10^8^ and the LOD of 10^−8^ M. The enhancement reproducibility fluctuated in a relative standard deviation of 1.61%, dropping in a 10^−6^ M R6G solution. The sensitivity AEF values of this substrate to detect the dilute paraquat and acetylcholine analytes were 3.19 × 10^7^ and 7.5 × 10^7^, respectively, and the LOD values were 10^−7^ M.

A SERS substrate deposited at a 6 nm thickness on glass slides can be prepared by DC sputtering to achieve the characteristics of cost-effectiveness, the required stable deposition rate, high mass production rate, and good sensitivity. In addition, the SERS substrates can have a long lifetime of more than forty weeks if stored in a vacuum or moisture-proof environment. These promising results indicate that the fabricated SERS substrate can be a candidate solution to apply to biomolecules, food safety, and environmental pollutants.

## Figures and Tables

**Table 1:** The thicknesses and their relative deposition times for Samples A–F.

Sample	A	B	C	D	E	F
Thickness (nm) *	3.0	4.5	6.0	7.6	9.1	12.1
Deposition time (s)	79	118	157	196	236	314

* The thickness recorded by a calibrated quartz crystal monitor.

**Table 2:** The grain sizes and hotspots for Samples A–E.

Sample	A	B	C	D	E	F
Grain size (nm) *	18.8 ± 4.5	25.3 ± 5.1	31.9 ± 8.4	42.4 ± 8.3	51.9 ± 10.2	-
Hotspot (nm)	5.30 ± 0.90	5.26 ± 0.98	5.29 ± 0.94	5.32 ± 0.93	5.37 ± 0.90	-

* Represented by maximum value ± FWHM/2 of Gaussian distribution.

**Table 3:** The crystalline scales of the as-deposited various AgNP layers.

Sample	Thickness (nm)	FWHM (nm)	Crystalline Size (nm)
A–E	3–9.1	--	--
F	12.1	1.413	6.21
G	18.2	1.074	8.18
H	24.2	0.879	9.98
I	30.3	0.814	10.78

**Table 4:** The compositions of Ag(0) and Ag(I) in Sample C stored in the different environments.

Sample	FWHM of Ag3d_5/2_	Ag(0) (at%)	Ag(I) (at%)
As-deposited	0.628	90.7	9.3
Vacuum desiccator (up to forty weeks)	0.632	78.4	21.6
Moisture-proof cabinet (up to forty weeks)	0.801	64.1	35.9
Air environment (up to forty weeks)	0.939	53.9	46.1

**Table 5:** The typical Raman shift of the R6G analyte.

Raman Shift (cm^−1^)	Assignment [36,37]
612	C–C ring in-plane bending in xanthene rings
771	C–H out-of-plane bending
1186	C–H in-plane bending in xanthene ring
1312	Hybrid mode (xanthene rings and NHC_2_H_5_ group)
1361	C–C stretching in xanthene ring
1509	C–C stretching in xanthene ring
1599	Hybrid mode (phenyl ring with COOC_2_H_5_
1646	C–C stretching in xanthene ring

**Table 6:** The S/B ratios of Samples A–F.

Sample	A	B	C	D	E	F
**S/B ratio**	4.63	3.15	2.64	1.94	2.18	-

**Table 7:** A comparison of the R6G LODs detected on a single silver SERS substrate deposited by sputtering methods in several studies over the last three years.

Material/Substrate	LOD of R6G (M)	Year of Publication	Reference
Ag/Si	10^−4^	2019	[55]
Ag/SiO_2_	10^−6^	2019	[56]
Ag/Polyester	10^−6^	2021	[57]
Ag/PMMA	10^−6^	2020	[58]
Ag/ZnO	10^−6^	2019	[59]
Ag/NiFe/cicada wing	10^−7^	2021	[60]
Ag/glass slide	10^−8^	2022	This study

## Data Availability

The data are included in the article.
